# Temperature Depended Role of *Shigella flexneri* Invasion Plasmid on the Interaction with *Acanthamoeba castellanii*

**DOI:** 10.1155/2012/917031

**Published:** 2012-02-28

**Authors:** Amir Saeed, David Johansson, Gunnar Sandström, Hadi Abd

**Affiliations:** Division of Clinical Microbiology, Department of Laboratory Medicine, Karolinska Institute and Karolinska University Hospital Huddinge, 14186 Stockholm, Sweden

## Abstract

*Shigella flexneri* is a Gram-negative bacterium causing the diarrhoeal disease shigellosis in humans. The virulence genes required for invasion are clustered on a large 220 kb plasmid encoding type three secretion system (TTSS) apparatus and virulence factors such as adhesions and invasion plasmid antigens (Ipa). The bacterium is transmitted by contaminated food, water, or from person to person. *Acanthamoebae* are free-living amoebae (FLA) which are found in diverse environments and isolated from various water sources. Different bacteria interact differently with FLA since *Francisella tularensis, Vibrio cholerae, Shigella sonnei*, and *S. dysenteriae* are able to grow inside *A. castellanii*. In contrast, *Pseudomonas aeruginosa* induces both necrosis and apoptosis to kill *A. castellanii*. The aim of this study is to examine the role of invasion plasmid of *S. flexneri* on the interaction with *A. castellanii* at two different temperatures. *A. castellanii* in the absence or presence of wild type, IpaB mutant, or plasmid-cured strain *S. flexneri* was cultured at 30°C and 37°C and the interaction was analysed by viable count of both bacteria and amoebae, electron microscopy, flow cytometry, and statistical analysis. The outcome of the interaction was depended on the temperature since the growth of *A. castellanii* was inhibited at 30°C, and *A. castellanii* was killed by invasion plasmid mediated necrosis at 37°C.

## 1. Introduction

*Shigella flexneri* is a Gram-negative bacterium and a human intestinal pathogen, causing the diarrhoeal disease bacillary dysentery (shigellosis), which is a worldwide health problem [1–4]. Shigellosis is characterized by invasion of, massive inflammation in, and destruction of the colonic mucosa. The genes required for invasion are clustered on a large 220 kb invasion plasmid that encodes the type three secretion system (TTSS) apparatus and the effectors' proteins, which implicate in the mechanism of characteristic cellular invasion of *Shigella* virulence such as adhesions and invasion plasmid antigens (Ipa) [5–7].

 It is well known that *S. flexneri* utilises TTSS to invade the epithelial layer of the colonic mucosa [7–9]. Infection of the macrophage and epithelial cells has been shown to be the responsible for TTSS effector Ipa proteins [10–12]. The bacteria escape from the phagocytic vacuoles shortly after entry into infected cells, but the outcome of the invasion process depends on the targeted cell [13]. In macrophages, IpaB activates series of reactions, which triggers the apoptosis of the macrophages. Unlike the situation in macrophages, the invasion of epithelial cells does not lead to apoptosis. Infected epithelial cells remain viable for many hours after infection [13].

*Shigella* is transmitted by contaminated food, water, or from person to person. Therefore, it is most common in areas with improper sanitation and personal hygiene and lack of a safe supply for drinking water [3, 14].

 Acanthamoebae are ubiquitous free-living amoebae that are distributed worldwide, living in diverse environments where different bacteria can be found, and Acanthamoeba species are isolated from various water sources [15]. Different bacteria interact differently with Acanthamoebae. Researchers have shown that many bacteria such as *Francisella tularensis*, *Vibrio cholerae*, *Shigella sonnei*, and* S. dysenteriae *[16–18] are able to grow inside *A. castellanii*. In contrast, *Pseudomonas aeruginosa* was found to kill *A. castellanii* since TTSS effectors proteins ExoS, ExoT, ExoU, and ExoY induced both necrotic and apoptotic killing [19].

It is known that *S. flexneri* infect macrophage and cause apoptosis by the effect of IpaB protein encoded by the invasion plasmid. The aim of the present study is to examine the role of invasion plasmid and IpaB protein of *S. flexneri* during the interaction with *A. castellanii* at two different temperatures.

## 2. Material and Methods

### 2.1. Bacterial Strains and Growth Conditions

*S. flexneri *serogroup 5a strains, M90T and BS176, are wild type and its virulence plasmid-cured mutant strain [9]. *S. flexneri* SF620 strain is an avirulent derivative of M90T that contains a nonpolar deletion of the *ipaB *gene [20]. Sansonetti et al., 1986, verified the presence and absence of the plasmid since the wild type infected >95% of HeLa cells and the plasmid cured strain failed to infect the Hela cells [9]. Moreover, Menard et al., 1993, found that the wild type M90T expressed IpaB protein compared to the nonpolar *IpaB* deletion mutant SF620 that failed to express the IpaB protein [20]. The strains were grown aerobically in Luria-Bertani medium at 37°C to an absorbance of 0.4 to 0.6 at 600 nm.

### 2.2. Amoeba Strain and Growth Conditions

*Acanthamoeba castellanii* (ATCC 30234) was obtained from the American Type Culture Collection, Manassas, VA, USA. *A. castellanii* was grown stationary at 30°C to a final concentration of 2 × 10^6^ cells/mL in ATCC medium no. 712 (ATCC).

### 2.3. Cocultivation Assay Conditions

The cocultivation assay was based on a previous method [17]. Briefly, cocultivation of each *Shigella *strain and *A. castellanii* was incubated in NUNC tissue culture flasks (75 cm^2^) purchased from VWR International (Stockholm, Sweden) filled with 50 mL ATCC medium 712 containing a concentration of 2 × 10^5^ cells/mL of *A. castellanii *and 2 × 10^6^ cells/mL of each *Shigella* strain and incubated at 30°C and 37°C until the end of experiments.

### 2.4. Microscopy Analysis

Culture samples were withdrawn for analysis at different time intervals. The numbers of live *S. flexneri *were determined by viable counts. The numbers of live *A. castellanii* cells were determined by using Erythrosine B stain (ATCC), which stained dead amoebae only. Briefly, 100 *μ*L of Erythrosine B solution were added to a 100 *μ*L cell suspension of *A. castellanii* in the absence or presence of *S. flexneri*. The unstained (live) cells of *A. castellanii* were counted in a Bürker chamber (Merck Eurolab, Stockholm, Sweden) under a light microscope (Carl Zeiss, Stockholm, Sweden) within 10 min after erythrosine staining.

To study morphology of *A. castellanii* cell in absence and presence of *S. flexneri* by electron microscope (EM), positive controls for apoptosis and necrosis as well as negative control in addition samples from amoebae cocultivated with *S. flexneri *strains were prepared. Cells of *A. castellanii* at 10^6^ cells/mL in ATCC medium no. 712 were incubated with 50 mM hydrogen peroxide (Merck) for 48 h at 37°C to induce necrosis and with 50 *μ*g/mL Actinomycin D (Sigma, Stockholm, Sweden) for 4 h at 37°C to induce apoptosis as positive controls. Cells of *A. castellanii* were incubated under the same conditions as negative control. The controls were centrifuged for 10 min at 300 ×g in Labofuge GL centrifuge (VWR International). After centrifugation, the pellets were washed in PBS.

For EM analysis, 5 mL of *A. castellanii* in absence or presence of *S. flexneri* cultivated at 30°C and 37°C after 1 day cultivation were centrifuged for 10 min at 300 ×g in Labofuge GL centrifuge (VWR International). After centrifugation, the pellets were washed in PBS. The pellets of the controls and *A. castellanii* in absence or presence of *S. flexneri* were fixed in 2.5% glutaraldehyde in 0.1 M sodium cacodylate buffer pH 7.3, with 0.1 sucrose and 3 mM CaCl_2_ for 30 min at room temperature. Samples were washed in sodium cacodylate buffer and postfixed in 2% osmium tetroxide in the same buffer for 1 h. The samples were centrifuged, dehydrated, and embedded in Epoxy resin, LX-112. The embedded samples were cut into ultrathin sections, put on grids, and stained with uranyl acetate and lead citrate. Sections were examined with a transmission electron microscope. A hundred cells was counted of each alone and cocultivated *A. castellanii* with wild type or plasmid-cured *S. flexneri* strain. The percentage of apoptotic, necrotic, and normal amoeba cells was determined by dividing number of each apoptotic, necrotic, and normal amoeba cell separately by the total number of all amoeba cells multiplied by 100.

### 2.5. Flow Cytometry

Fluorescence activated cell sorting (FACS) analysis of Propidium iodide (PI) staining was used for quantification of necrosis. Briefly, amoebae in the absence and presence of *S. flexneri* were cultivated for 24 h, samples of the amoebae washed twice in PBS and 100 *μ*L with 5 × 10^5^ amoebae/mL of each sample were incubated in dark for 10 min at room temperature with 10 *μ*g/mL PI reagent (Roch diagnosis GmbH, Mannheim Germany). Finally 500 *μ*L PBS was added to each sample and the samples were analyzed using a FACSCalibur flow cytometer (BD Biosciences, NJ, USA). It was clear from earlier trails that background from PI staining was not elevated in diluted samples or in washed samples. The dilution method was recommended by the manufacture and used to perform FACS analysis in this study.

### 2.6. Statistical Analysis

The Student *t*-test and *χ* ^2^ test were used to test for statistically significant differences in growth of *A. castellanii* in the absence or presence of *S. flexneri* strains.

## 3. Results

### 3.1. Growth and Survival of Wild Type *S. flexneri* in the Presence or Absence of *A. castellanii* at 30°C

**Figure 1:**
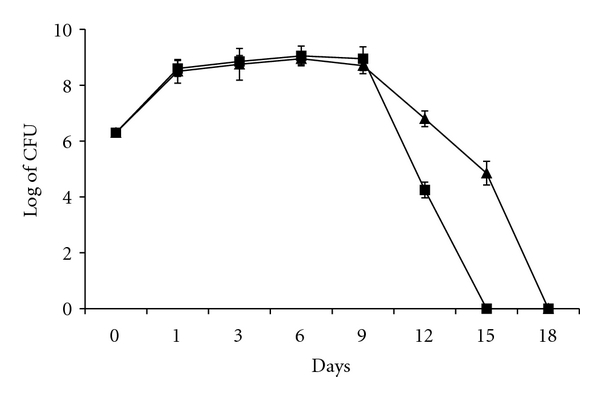
Viable counts of *S. flexneri* at 30°C. Alone cultivated bacteria (■) and co-cultivated with *A. castellanii* (▲). The points are mean ± SD of three samples.

To examine growth of *S. flexneri* in the presence or absence of *A. castellanii* at 30°C, viable counts of alone and cocultivated *S. flexneri* with *A. castellanii* were performed. It was found that both alone and cocultivated bacteria increased 100 folds on day 1, under the log-growth phase. The stationery growth phase continued from day 2 to day 9 and then the decline phase finished on day 15 for alone-cultivated *S. flexneri* and on day 18 for cocultivated bacteria. However, *A. castellanii* enhanced survival of cocultivated bacteria, which survived longer since the alone and cocultivated *S. flexneri* become nondetectable by viable count assay on days 15 and 18, respectively (Figure 1).

### 3.2. Growth of *A. castellanii* in the Presence or Absence of *S. flexneri* at 30°C

**Figure 2:**
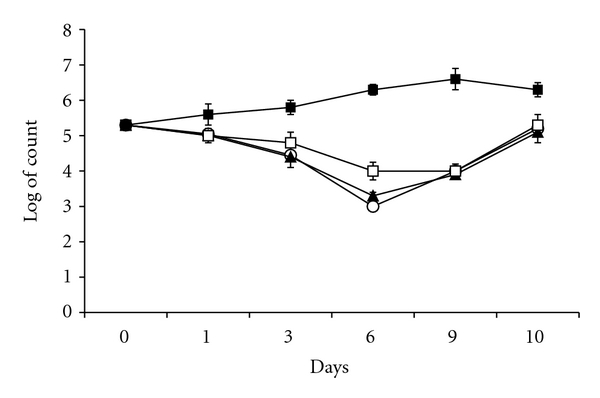
Viable counts of amoeba cells at 30°C. Alone cultivated amoebae (■), co-cultivated with wild type *S. flexneri* (○), with IpaB mutant (▲), and with plasmid cured (□) strains. The points are mean ± SD of three samples.

Number of viable *A. castellanii *in absence of *S. flexneri* increased from 2 × 10^5^ cell/mL on day 0 to 2 × 10^6^ cell/mL from day 6 to day 10 (Figure 2).

Growths of cocultivated *A. castellanii* (Figure 2) were inhibited under log- and stationery growth phases of the bacteria as shown in Figure 1. The number of viable *A. castellanii* cocultivated with wild type, *IpaB* mutant, or plasmid cured strain *S. flexneri* was 2.0 × 10^5^ cell/mL on day 0 and decreased to 1.0 × 10^4^ cell/mL on day 9 (Figure 2) under log- and stationery growth phases of the bacteria. Interestingly, the number of viable cocultivated amoebae reached their initial numbers (2.0 × 10^5^ cell/mL) when the bacterial decline growth phase started on day 10.

### 3.3. Growth and Survival of *A. castellanii* in the Presence or Absence of *S. flexneri* at 37°C

*A. castellanii* in the presence of wild type, IpaB mutant, and plasmid-cured* S. flexneri* strains at 37°C did not grow but survived 0.5, 2.0, and 6.0 days, respectively, compared to *A. castellanii* in absence of the wild type *S. flexneri*, which grew from 2 × 10^5^ cell/mL on day 0 to 4 × 10^5^ cell/mL on day 3 and survived at this rate until end of the experiment on day 7.

**Figure 3:**
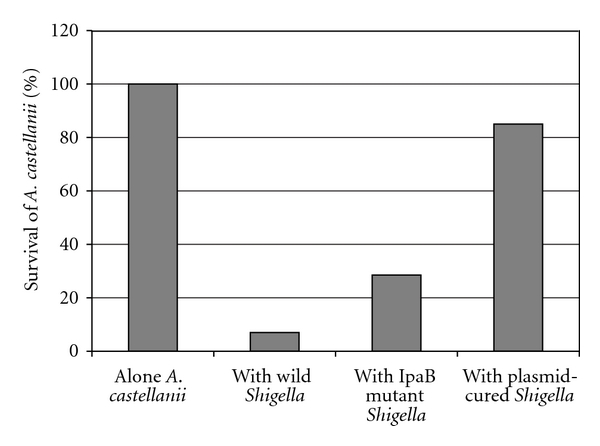
Survival of alone and cocultured *A. castellanii*. Data indicating mean survival time from three independent experiments expressed in percentage.

However, to estimate percentage of survival time of *A. castellanii* cocultivated with each *S. flexneri* strain, alone *A. castellanii* survived 7 days, which considered 100%. The days that cocultivated amoeba survived with each *S. flexneri* strain are divided by 7 to determine percentage of the survival. Survival times % of *A. castellanii* cocultivated with wild type *S. flexneri*, with IpaB mutant, and with plasmid-cured strains were 7%, 28.5%, and 85%, respectively (Figure 3).

The statistical analysis by *χ* ^2^ test showed that survival time of alone-cultivated *A. castellanii* compared to the survival of cocultivated with wild type *S. flexneri* was very highly significant (*P* < 0.0000001), but survival of alone-cultivated *A. castellanii* compared to that of cocultivated with plasmid-cured *S. flexneri* was not statistically significant (*P* = 0.88). However, the survival time of alone-cultivated *A. castellanii* compared to the survival of cocultivated with *IpaB* mutant *S. flexneri* was highly significant (*P* < 0.00001).

On the other hand, survival of *A. castellanii* cocultivated with wild type *S. flexneri *compared to that of *A. castellanii* cocultivated with plasmid-cured *S. flexneri* was highly significant (*P* < 0.0001).

### 3.4. Visualisation of Bacterial Effect on Amoebae at 30°C

To study effect of *S. flexneri* on *A. castellanii*, electron microscopy was used to analyse samples of alone-cultivated *A. castellanii* as well as cocultivated with wild type or with plasmid cured *S. flexneri* strains.

Electron microscopy estimates percentage of individual apoptotic, necrotic as well as normal cells depending on morphological alteration of nucleus and plasma membrane. Chromatin condensation and reduced size of the nucleus are typical features of apoptosis compared to disruption of plasma membrane and ghost shape of the cell, which are typical features of necrosis.

**Figure 4:**
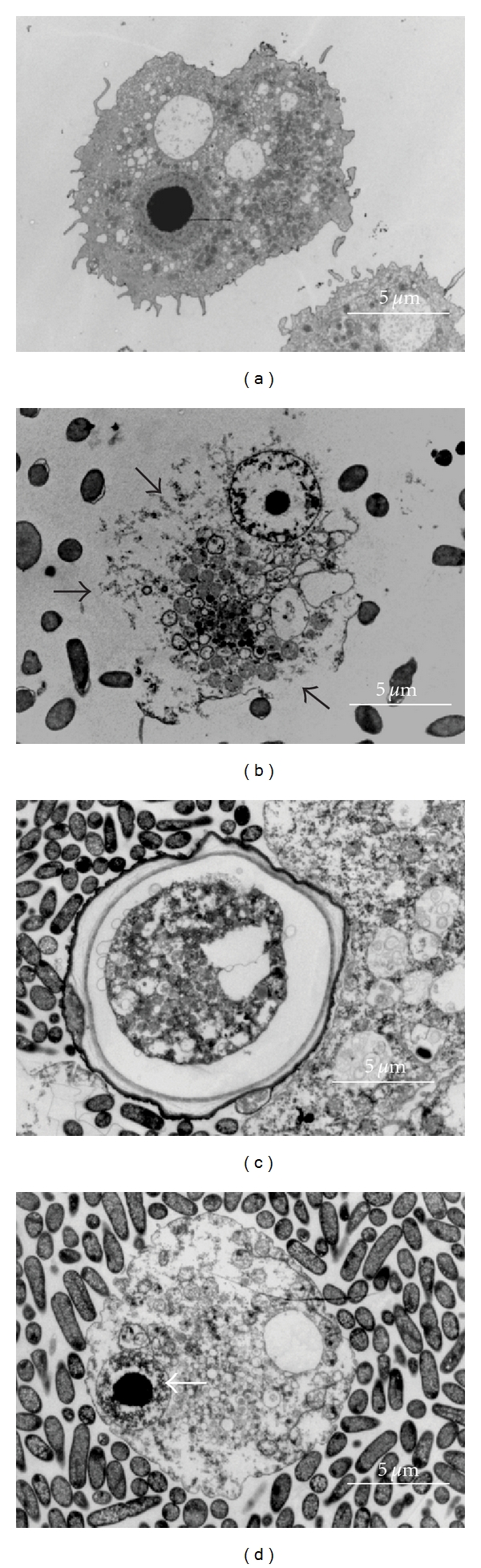
Electron microscopy at 30°C, black arrows point out necrosis and white apoptosis. *A. castellanii* trophozoite cultivated alone (a), *A. castellanii* trophozoite cocultivated with wild type *S. flexneri* showing necrosis (b), *A. castellanii* cyst cocultivated with plasmid-cured *S. flexneri* (c), and *A. castellanii* trophozoite cocultivated with plasmid-cured *S. flexneri* showing apoptosis (d).

 The analysis at 30°C found that alone-cultivated *A. castellanii* showed 4 ± 0.5% apoptosis, 2 ± 0% necrosis, and 94 ± 1.4% normal cells. Compared to alone-cultivated *A. castellanii* (Figure 4(a)), amoebae cocultivated with wild type *S. flexneri *showed 86 ± 3% necrosis (Figure 4(b)), 6 ± 1% apoptosis, and 8 ± 1 normal cells. Amoebae cocultivated with plasmid-cured *S. flexneri* showed 26 ± 2.8% necrosis, 72 ± 0  normal cells, which were cysts (Figure 4(c)), and 2 ± 1% apoptosis (Figure 4(d)).

### 3.5. Visualisation of Bacterial Effect on Amoebae at 37°C

**Figure 5:**
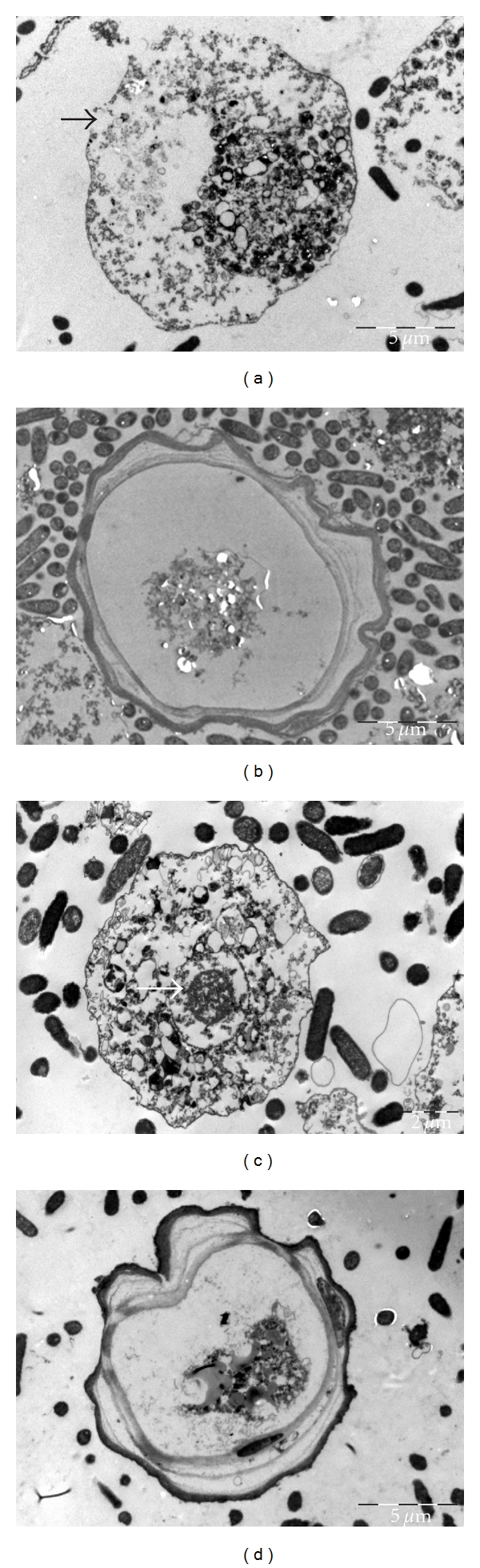
Electron microscopy at 37°C, black arrows point out necrosis and white apoptosis. *A. castellanii* trophozoite cocultivated with wild type *S. flexneri* showing necrosis (a), *A. castellanii* cyst cocultivated with wild type *S. flexneri* (b), *A. castellanii* trophozoite cocultivated with plasmid-cured *S. flexneri* showing apoptosis (c), and *A. castellanii* cyst cocultivated with plasmid-cured *S. flexneri* (d).

Alone-cultivated *A. castellanii* showed 3 ± 1% apoptosis, 5 ± 1% necrosis, and 92 ± 1% normal cells. Amoebae cocultivated with wild type *S. flexneri *showed 93 ± 1% necrosis (Figure 5(a)), 4 ± 1% apoptosis, and 3 ± 1% normal cells. All the normal cells were cysts (Figure 5(b)). Amoebae cocultivated with plasmid-cured *S. flexneri* showed 90 ± 5% necrosis, 6 ± 1% apoptosis (Figure 5(c)), and 4 ± 1% normal cells. Again, all the normal cells were cysts (Figure 5(d)).

### 3.6. *S. flexneri * Induces Rapid Membrane Damage to *A. castellanii*

Flow cytometry measures fluorescence intensity of a cell population in this case stained with PI which is a DNA-binding dye not able to penetrate the cell membrane and thus only stains cells with disrupted membrane integrity.

Analysis at 30°C showed that the percentages of normal and necrotic *A. castellanii* in absence of *S. flexneri* stains were 88.2% and 11.7%, respectively. The percentages of necrotic amoeba cells cocultivated with *S. flexneri* wild type, IpaB mutant and plasmid-cured strains were 99.8%, 99.7%, and 83.3%, respectively. Moreover, percentage of normal amoeba cells cocultivated with plasmid-cured strain was 15.6% (Data not shown).

**Figure 6:**
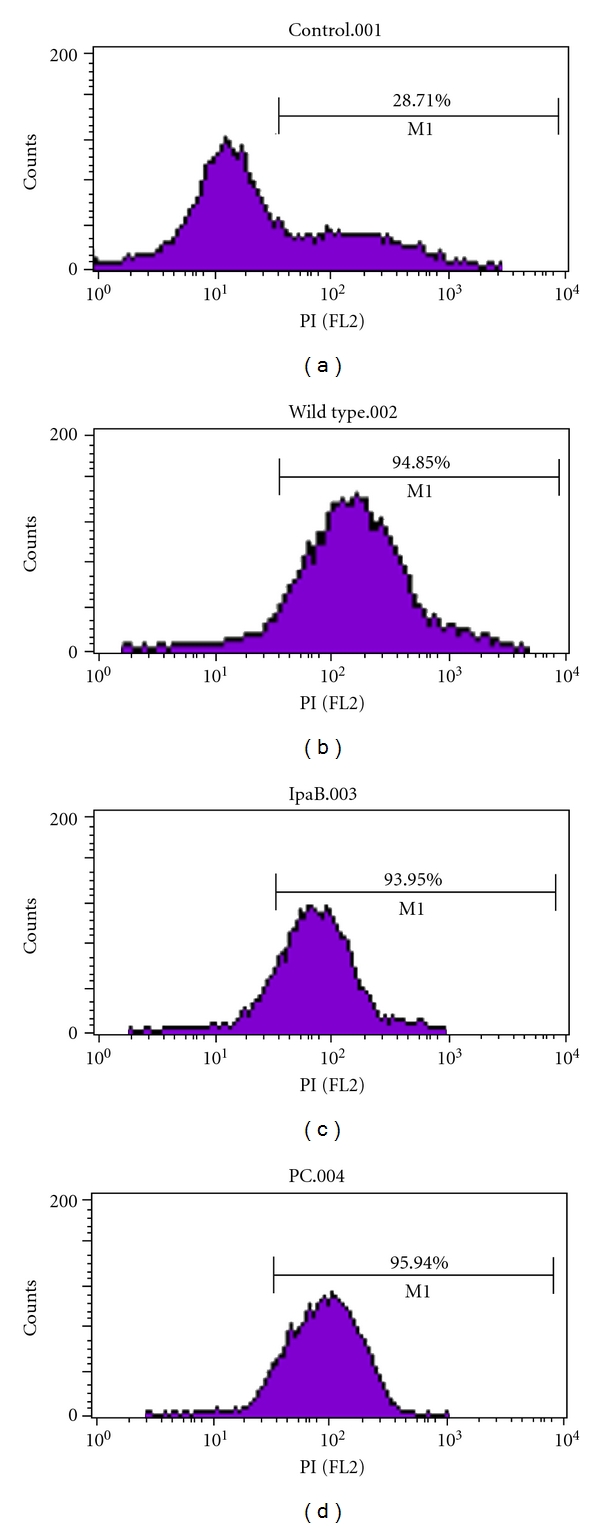
Necrosis analysis by flow cytometry at 37°C. *A. castellanii* alone (a), *A. castellanii* co-cultivated with wild type *S. flexneri* (b), *A. castellanii* co-cultivated with IpaB mutant *S. flexneri* (c) and *A. castellanii* co-cultivated with plasmid-cured *S. flexneri* (d).

Analysis at 37°C showed that the percentages of normal and necrotic *A. castellanii* in absence of *S. flexneri* stains were 71.3% and 28.7%, respectively. The percentages of necrosis in amoeba cells cocultivated with *S. flexneri* wild type, IpaB mutant and plasmid-cured strains were 94.85%, 93.70%, and 94.82%, respectively (Figure 6).

## 4. Discussion

Shigellosis is a global health problem mostly affecting young children [3]. *S. flexneri* is a waterborne pathogen, it may interact with *A. castellanii *present in water and this may prime the *S. flexneri* for infection of the host cell. In this study, role of the IpaB protein and the invasion plasmid on the interaction of *S. flexneri* with *A. castellanii* was examined. It is thought that any findings may uncover a better understanding of the pathogenesis of *S. flexneri* and contribute to improved treatment of the infection.

The results showed that wild type *S. flexneri* at 30°C inhibited growth of *A. castellanii* to a rate that is lower than alone-grown amoebae (*P* < 0.001), but the effect did not result in killing of the amoebae. However, at 37°C it was found that the wild type, *IpaB* mutant, and plasmid cured strains of *S. flexneri *killed *A. castellanii*. In this context, it is well known that the invasive property of *S. flexneri* depended on the 220-kb plasmid, which is strongly temperature regulated. Maurelli et al. found that virulent strains of *S. flexneri*, *S. sonnei*, and *S. dysenteriae* at 37°C were invasive, and usually greater than 90% of Henle 407 human intestinal epithelial cells could be seen to have bacteria in the cytoplasm. However, these virulent *Shigella* strains at 30°C failed to invade the Henle cells, which were also free of adherent bacteria. Interestingly, it was found that growth of the virulent strains of *S. flexneri*, *S. sonnei*, and *S. dysenteriae* at 30°C inhibited expression of invasiveness of these strains for Henle cells [21]. This finding may explain why growth of the cocultivated amoebae with *S. flexneri* is inhibited at 30°C and the amoebae are killed at 37°C.

Our result showed that wild type, *IpaB* mutant and plasmid-cured strains of *S. flexneri *killed *A. castellanii* in 0.5, 2.0, and 6.0 days, respectively. Interestingly, the statistical analysis found that survival of alone-cultivated amoebae compared to that of cocultivated with wild type *S. flexneri* was very highly significant (*P *value of *χ* ^2^ test was <0.0000001), but survival of alone-cultivated amoebae compared to that of cocultivated with plasmid-cured *S. flexneri* was not statistically significant (*P* = 0.88). The very highly statistical significant explains the important role of invasion plasmid on amoebae killing and as a potent virulence factor for *S. flexneri*.

However, the survival time of alone-cultivated *A. castellanii* compared to the survival of the cocultivated with *IpaB* mutant *S. flexneri* was highly significant (*P* < 0.00001), which suggests clearly presence of other *Ipa *genes rather than *IpaB* such as *IpaC* and *IpaD*. On the other hand, the significant different survival time of amoebae cocultivated with wild type *S. flexneri *compared to that of amoebae cocultivated with plasmid-cured *S. flexneri* (*P* < 0.0001) suggests strongly presence of gene/genes for other virulence factors not located in the invasion plasmid but in the chromosome. The other plasmid-located TTSS genes (rather than *IpaB*) and chromosome-located TTSS genes that will be activated at 37°C may affect the outcome of interaction between *S. flexneri* and FLA.

The aim of this study was to examine the role of invasion plasmid and IpaB protein of *S. flexneri* during the interaction with *A. castellanii* at two different temperatures. Anyhow, role of other plasmid-located and chromosome-located TTSS genes in interaction between *S. flexneri* and FLA will be subjects to next study.

The result has shown that *S. flexneri* inhibits growth of the amoebae when invasion plasmid was not activated at 30°C (Figure 2). The inhibition occurred while the bacteria were in log- and stationary growth phases (Figure 1). However, the amoebae returned to its initial viable number when the bacteria entered the decline phase (Figures 2 and 1). This may explain that the bacteria produce virulence factors not mediated by the invasion plasmid and these factors are not able to kill the amoebae but can inhibit growth of the amoebae. In this context, it was shown that *A. castellanii* cocultivated with *S. sonnei* or with *S. dysenteriae* was not inhibited but grew at 30°C from 10^5^ cell/mL to 10^6^ cell/mL while these *Shigella* species killed the amoebae at 37°C [18]. There are differences between *Shigella* species (*S. dysenteriae *and *S. sonnei*) as studied in [18] and *S. flexneri* studied in the current study. The log- and stationary growth of both alone and cocultivated *S. flexneri* lasted 9 days. However, the log- and stationary growth phases of both alone-cultivated *S. dysenteriae* and *S. sonnei* lasted 6 days compared to that of cocultivated *S. dysenteriae* and *S. sonnei* lasted 18 and 15 days, respectively. Moreover, numbers of alone and cocultivated *S. flexneri* were nondetectable by viable count method at day 15 and day 18, respectively. Numbers of alone-cultivated *S. dysenteriae* and *S. sonnei* become nondetectable by viable count method at day 18 and day 15, respectively. Interestingly, viable counts of each *S. dysenteriae* and *S. sonnei* cocultivated with amoebae were 10^8^ CFU/mL at day 21. The important difference is that both *S. dysenteriae* and *S. sonnei* did not inhibit growth of the amoebae at 30°C compared to *S. flexneri* that inhibited growth of the amoeba. Surprisingly, *S. dysenteriae*, *S. sonnei*, and *S. flexneri* killed the amoeba population by induction necrosis at 37°C. This finding may indicate that *S. sonnei* and *S. dysenteriae* are less virulent against the amoeba at 30°C, which may allow these strains to grow and survive inside *A. castellanii* [18].

Abd et al. reviewed in [19] that cell death is classified by either apoptosis or necrosis. Distinguishing features of apoptosis are chromatin condensation (pyknosis) and DNA fragmentation (karyorrhexis), and features of necrosis are plasma membrane disruption and nuclear disintegration (karyolysis). The apoptotic process in mammalian cells, as well as in unicellular eukaryotes such as *Acanthamoeba polyphaga*, and *Dictyostelium discoideum*, depends on activation of caspases and mitochondrial outer membrane permeabilisation. Microbial pathogens improve their ability to persist in infected hosts by competing with host defence systems. Extracellular bacteria, such as *Corynebacterium diphtheriae*, *Pseudomonas aeruginosa*, and *Bacillus anthracis*, are sensitive to phagocytosis; therefore, they may benefit from killing macrophages before they are ingested. Moreover, infection of epithelial cells by *P. aeruginosa* resulted in a surface upregulation of CD95 and CD95 ligand. The upregulation depends on the function of the TTSS of *P. aeruginosa*. Binding of CD95 by the CD95 ligand upon upregulation induces the activation of caspases 8 and 3, and the release of mitochondrial cytochrome c, which leads to apoptosis. On the other hand, facultative intracellular bacteria may employ different strategies to prevent cell death during their replication. However, macrophage apoptosis is an end stage for bacterial proliferation [19].

The cell death is triggered by different biochemical pathways and it is involved in the interaction between host cells and bacterial pathogens [22]. *Shigella* species have been reported to be able to induce apoptosis in macrophages by secreting effector proteins such as *IpaB* protein into the host cell by TTSS [23–27]. Moreover, Monocyte-derived macrophages infected with *S. flexneri* undergo a rapid cytolytic event resulted in cell death (necrosis) characterised by rupture of the plasma membrane, cell swelling, disintegration of the cellular ultra structure, and generalized karyolysis [28].

It has been reported that TTSS proteins of the extracellular bacterium *P. aeruginosa* induced apoptosis to kill macrophages [29] and it induced both apoptosis and necrosis in *A. castellanii* [19]. While, the facultative intracellular bacterium, *L. pneumophila* did not induce apoptosis but necrosis in *A. polyphaga* [30].

Different methods are used in this paper such as viable count assay, electron microscopy, and flow cytometry. These methods estimate absolute number of the individual viable cells, percentage of individual apoptotic, necrotic as well as normal cells, and percentage of fluorescence intensity emitted by the IP stained cell population, respectively. Regarding the methodology, viable count determines number of living amoebae in 1 *μ*L cell suspension compared to total number of cells in pellet of 2-3 mL cell suspension by electron microscopy and to percentage of the fluorescence intensity emitted by cell population by FACS, which examines 60 *μ*L/min.

In addition to the methodological considerations, there are biological considerations regarding growth and encystation of *A. castellanii* that may play a role in the interaction. *A. castellanii* trophozoites have a wide growth temperature range of 12°C to 45°C, but the optimal growth temperature is 30°C [31]. Our practical experience showed that *A. castellanii* grew highest up to 2.0 × 10^6^ cell/mL at 30°C and up to 4.0 × 10^5^ cell/mL at 37°C. That is why the percentage of the normal *A. castellanii* in absence of *S. flexneri* was 88.2% at 30°C and decreased to 71.3% at 37°C, compared to the percentage of the necrotic* A. castellanii* in absence of *S. flexneri*, which was 11.7% at 30°C and increased to 28.7% at 37°C, respectively.

Whatever, despite these different analysis methods did not give same exact percentages of necrosis, these methods showed that inhibition and killing of *A. castellanii* was due to the necrosis caused mostly by the invasion plasmid of *S. flexneri* under different temperatures 30°C and 37°C.

## 5. Conclusions

Findings in this paper together with findings of other researcher may conclude that *Shigella* has the ability to control the way it kills the host cells, as it clearly commands mechanisms that lead to either apoptosis or necrosis. Thus, such ability must play a crucial role in the interaction of *Shigella* with different host cells at different temperatures since invasion plasmid playing very important role.
